# A Promising Polymer Blend Electrolytes Based on Chitosan: Methyl Cellulose for EDLC Application with High Specific Capacitance and Energy Density

**DOI:** 10.3390/molecules24132503

**Published:** 2019-07-09

**Authors:** Shujahadeen B. Aziz, M. H. Hamsan, Ranjdar M. Abdullah, M. F. Z. Kadir

**Affiliations:** 1 Advanced Polymeric Materials Research Lab., Department of Physics, College of Science, University of Sulaimani, Qlyasan Street, Sulaimani 46001, Kurdistan Regional Government, Iraq; 2 Komar Research Center (KRC), Komar University of Science and Technology, Sulaimani 46001, Kurdistan Regional Government, Iraq; 3 Centre for Foundation Studies in Science, University of Malaya, Kuala Lumpur 50603, Malaysia

**Keywords:** Biopolymer blend electrolyte, XRD analysis, Morphology study, Impedance spectroscopy, TNM and LSV, cyclic voltammetry, EDLC fabrication, Specific capacitance, energy density

## Abstract

In the present work, promising proton conducting solid polymer blend electrolytes (SPBEs) composed of chitosan (CS) and methylcellulose (MC) were prepared for electrochemical double-layer capacitor (EDLC) application with a high specific capacitance and energy density. The change in intensity and the broad nature of the XRD pattern of doped samples compared to pure CS:MC system evidencedthe amorphous character of the electrolyte samples. The morphology of the samples in FESEM images supported the amorphous behavior of the solid electrolyte films. The results of impedance and Bode plotindicate that the bulk resistance decreasedwith increasing salt concentration. The highest DC conductivity was found to be 2.81 × 10^−3^ S/cm. The electrical equivalent circuit (EEC) model was conducted for selected samples to explain the complete picture of the electrical properties.The performance of EDLC cells was examined at room temperature by electrochemical techniques, such as impedance spectroscopy, cyclic voltammetry (CV) and constant current charge–discharge techniques. It was found that the studied samples exhibit a very good performance as electrolyte for EDLC applications. Ions were found to be the dominant charge carriers in the polymer electrolyte. The ion transference number (*t_ion_*) was found to be 0.84 while 0.16 for electron transference number (*t_el_*). Through investigation of linear sweep voltammetry (LSV), the CS:MC:NH_4_SCN system was found to be electrochemically stable up to 1.8 V. The CV plot revealed no redox peak, indicating the occurrence of charge double-layer at the surface of activated carbon electrodes. Specific capacitance (*C_spe_*) for the fabricated EDLC was calculated using CV plot and charge–discharge analyses. It was found to be 66.3 F g^−1^ and 69.9 F g^−1^ (at thefirst cycle), respectively. Equivalent series resistance (*R_esr_*) of the EDLC was also identified, ranging from 50.0 to 150.0 Ω. Finally, energy density (*E_d_*) was stabilized to anaverage of 8.63 Wh kg^−1^ from the 10th cycle to the 100th cycle. The first cycle obtained power density (*P_d_*) of 1666.6 W kg^−1^ and then itdropped to 747.0 W kg^−1^ at the 50th cycle and continued to drop to 555.5 W kg^−1^ as the EDLC completed 100 cycles.

## 1. Introduction

Typically, two types of host polymers are used in the preparation of solid polymer blend electrolytes (SPBEs) for energy storage devices: natural and synthetic polymers [1]. Most synthetic polymers, such as polyethylene, polyvinyl chloride, polymethylmetacrylate, polycarbonate and polyacrylonitrile, are non-biodegradable [2]. Non-biodegradable synthetic polymers generally give rise to the depletion of petroleum resources and the disposal issues [3]. Thus, to study the energy storage devices as well as reduce the plastic waste pollution, biopolymers can be used as the host polymer. Biopolymers are biodegradable polymers produced from natural resources. They often have unique properties, such as inexpensive, compatible with various solvents, abundance and good film forming [4,5]. Starch, cellulose, chitosan and carrageenan are the most commonly used biopolymers in the study of polymer electrolytes [6,7,8,9].

Chitosan (CS) is an amino polysaccharide extracted from the shell of crustaceans with repeating units of β-(1→4)2-amino-2-deoxy-d-glucose-(d-glucosamine) [10]. The introduction of methyl chloride or dimethyl sulfate to alkali based cellulose creates a modified biopolymer called methylcellulose (MC) with1,4 glycosidic bond [11]. Ions make a complexation with oxygen containing functional groups of the polymer host through dative bond. CS has functional groups with lone pair electrons, such as amine, hydroxyl and glycosidic bond groups, while methylcellulose has hydroxyl, glycosidic bond and mexthoxy groups for the conduction of ions [12]. Thus, through polymer blend method, a polymer blend host with more sites for ionic conduction process can be produced [13].

EDLC is an alternative energy storage device that is capable of replacing conventional chemical batteries. The mechanism of energy storage for EDLC involves non-Faradaic reaction. The addition of salt in the electrolyte produces positively charged ion (cations) and negatively charged ions (anions). As the EDLC is subjected to a working voltage, cations and anions will migrate to negative and positive electrodes, respectively [14]. As the EDLC is connected to a power source (charging), one of the electrodes becomes negative and the electric field building up at the electrode attracts cations and repels anions whilethe opposite action happens at the positive electrode. The intense electric field holds ions from the electrolyte and electrons from the electrode. This is called thedevelopment of charge double- layer where it stores the energy as potential energy [15]. On the other hand, during discharging process, the oppositeaction occurs.Unlike Faradaic capacitor or pseudocapacitor, EDLCs are reported to have higher power density, longer life cycle, better thermal stability, and higher reversibility; they arealso cheaper, safer and simpler tofabricate [16,17]. Activated carbon has been studied to achieve good compatibility with polymer electrolyte [18,19]. Good properties of activated carbon, such as high electrical conductivity, inexpensive and excellent chemical stability as well as large surface area, make it preferable for the EDLC application [20]. Recently, proton conducting polymer-based electrolytes attract the efforts of many researchers for the EDLC application. In this study, the electrical properties of CS-MC blend host doped with ammonium thiocyanate (NH_4_SCN) wereanalyzed. The highest conducting electrolyte was used as electrodes separator in EDLC. 

## 2. Experimental Method

### 2.1. Materials and Sample Preparation

In this work, chitosan (with high molecular weight of approximately 310,000–375,000 g/mol) and methyl cellulose as the raw materials were used and provided by Sigma-Aldrich (Darmstadt, Germany). For the preparation of polymer blend host based on CS:MC, 70 wt.% of CS and 30 wt.% MC were dissolved separately in 40 mL of 1% acetic acid at room temperature for 3 h. The CS:MC solution was then mixed and stirred for 2 hto obtain a homogeneous blend mixture. For the prepared mixture, different amounts of NH_4_SCN salt ranging from 10 to 40 wt.% in steps of 10% wereadded separately along with continuousstirring to prepare CS:MC:NH_4_SCN electrolytes. The electrolyte solutions were then cast in different Petri dishes and left to dry slowly at ambient temperature to form films. The films were transferred into a desiccator for further drying. This procedure produces solvent-free films. The polymer blend electrolyte samples were coded as CSMCB0, CSMCB1, CSMCB2, CSMCB3, and CSMCB4 for CS:MC incorporated with 0, 10, 20, 30, and 40 wt.% of NH_4_SCN, respectively.

### 2.2. Structural, Morphological and Impedance Characterizations

X-ray diffraction (XRD) analyses were carried out through a Siemens D5000 X-ray diffractometer (1.5406 Å). The scanning range of 2θ angle was from 10° to 80° with step size of 0.1°. XRD results were deconvoluted using Origin 9.0 software with a baseline correction and Gaussian function. A Hitachi SU8220 FESEM (Tokyo, Japan) was used to study the surface morphology of the blend electrolyte films at 500× magnification. Electrical impedance spectroscopy (EIS) for the samples was conducted via a HIOKI 3532–50 LCR Hi-TESTER in the frequency range of 50 Hz to 5 MHz. For this purpose, the samples were sandwiched in between two stainless steel electrodes.

### 2.3. TNM AND LSV Measurements

Transference number measurements (TNMs) were performed at room temperature using direct current (DC) polarization method by tracking the polarization current as a function time. A cell was polarized at 0.2 V using a V&A Instrument DP3003 digital DC power supply. To the test electrochemical stability of the cells, linear sweep voltammetry (LSV) was employed, using Digi-IVY DY2300 potentiostat at scan rate of 50 mV s^−1^. For both TNM and LSV, the cell consisted of the highest conducting polymer blend electrolyte (CSMCB4) sandwiched by two stainless steel electrodes.

### 2.4. EDLC Preparation 

**Scheme 1:**
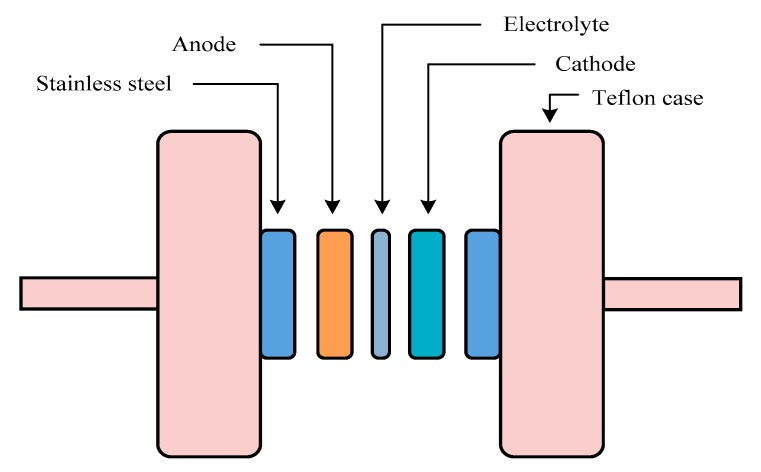
Diagram of the electrochemical double-layer capacitor (EDLC) cell.

The activated carbon electrodes for the EDLC cells were prepared by first dry mixing of activated carbon (3.25 g) with carbon black (0.25 g). The dry mixing process was carried out by using a planetary ball miller (XQM-0.4). Six metal balls were inserted into the chamber along with the powders. The powders were mixed at rotational speed of 500 r/min for 15 min. The powders were then added to a solution of 0.5 g of polyvinylidene fluoride (PVdF) in 15 mL of *N*-methyl pyrrolidone solvent. After the dissolution, the obtained homogeneous solution was coated on an aluminum foil and dried in an oven at 60 °C to form a highly conductive carbon-based electrode. The EDLC cells were then assembled with the obtained carbon electrodes and the highest conducting polymer blend electrolyte (CSMCB4) in a coin cell (CR2032). Digi-IVY DY2300 potentiostat was utilized to analyze cyclic voltammetry (CV) of the EDLC cells at 10 mV s^−1^ and charged up to 0.90 V. The charge–discharge profiles for the cells were tested using Neware battery cycler with current density of 0.4 mA cm^−2^. The schematic diagram of an EDLC cell is shown in Scheme 1.

## 3. Results and Discussion

### 3.1. Structural and Morphological Study

**Figure 1:**
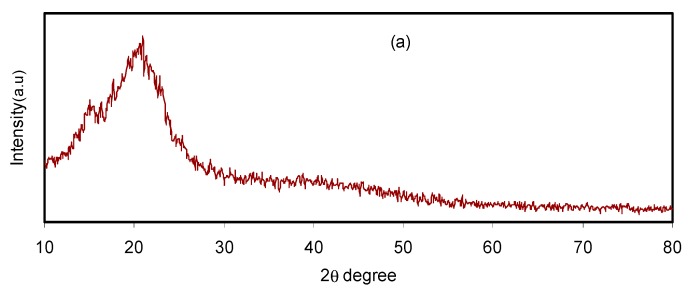

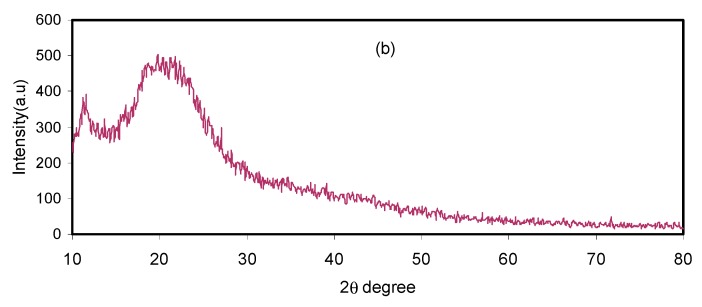
Pattern for: (**a**) pure CS; and (**b**) CS:MC films.

The diffractograms of the pure chitosan, chitosan:MC films are shown in Figure 1a,b. In reference to Hamsan et al. [21], MC possesses crystalline peaks at 2θ = 10.2°, 16.3°, 19.8° and 22.0° while Kadir and Hamsan [22] reported that crystalline peaks for CS can be observed at 2θ = 11.6°, 17.7°, 20.0° and 23°. Hence, the deconvolution of XRD results in this work was based on these crystalline peaks. The results shown in Figure 1 are due to the existence of inter- and intra-hydrogen bonding among the functional groups of individual monomers and between the chains [23,24,25,26,27]. It is interesting to notice that the XRD pattern of the CS:MC system shows only two concave peaks (see Figure 1b). These broad peaks imply that the CS:MC blend composed of complete amorphous structure [27,28,29]. Usually, the concept of physical blending of two or more polymers has been made as a strategy to suppress the crystallinity and thus enhance the conductivity. Polymer blends are physical mixes of at least two structurally different kinds of polymers, which interact through secondary forces with non-covalent bonding [30].

**Figure 2:**
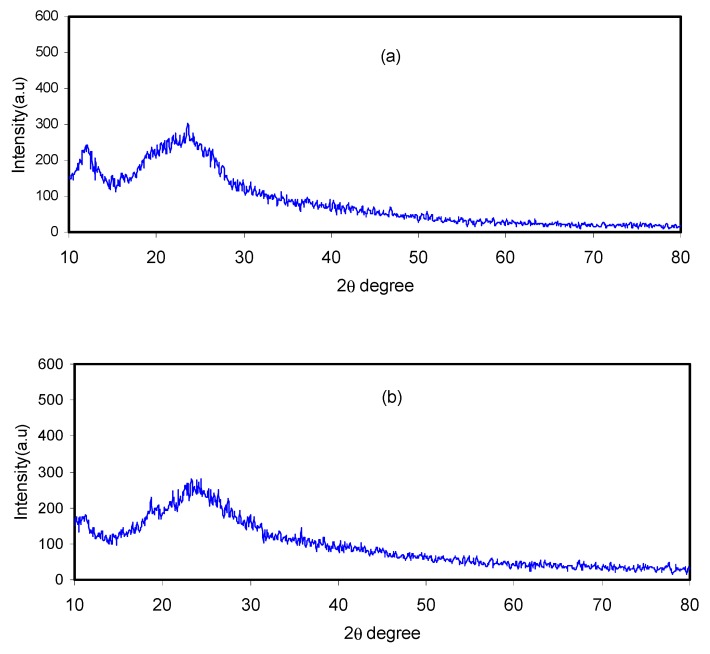
For: (**a**) CSMCB2; and (**b**) CSMCB4 films.

**Figure 3:**
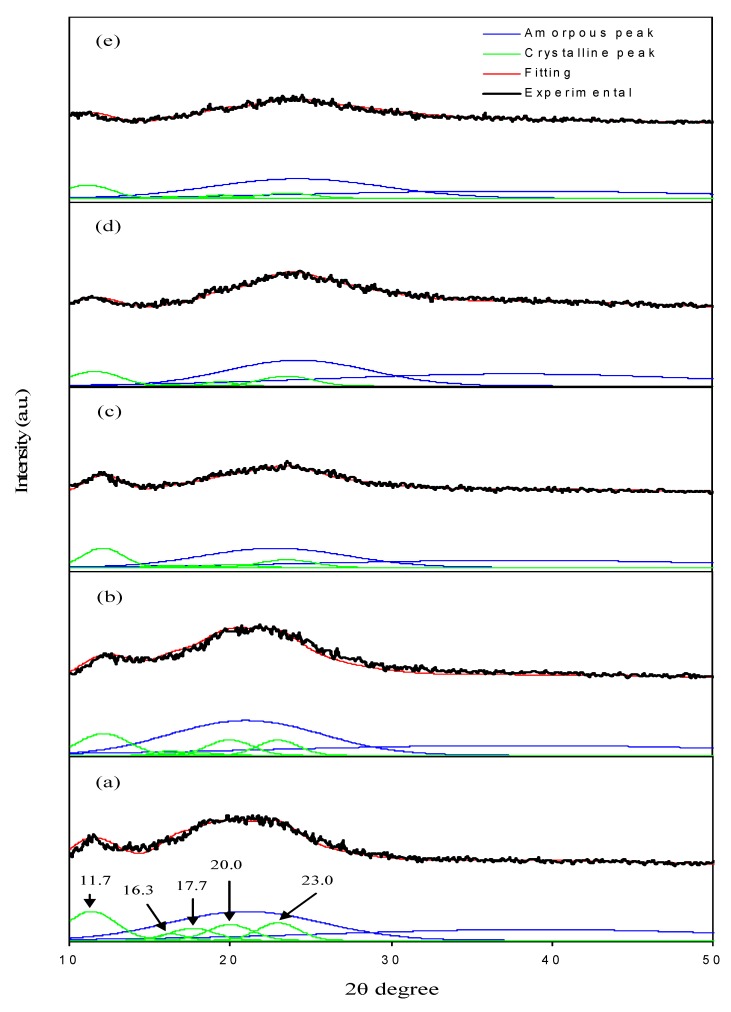
XRD pattern for: (**a**) CS:MCB0; (**b**) CSMCB1; (**c**) CSMCB2; (**d**) CSMCB3; and (**e**) CSMCB4 films.

The XRD pattern for selected blend electrolyte samples are shown in Figure 2a,b. It is obvious that, with addition of 20 wt.% of inorganic NH_4_SCN salt into CS:MC matrix, the peak intensity of theCS:MC was drastically decreased, as shown in Figure 2a. This indicates that the crystalline region of polymer blend matrix was reduced [24]. More clearly, the addition of 40 wt.% of NH_4_SCN salt into CS:MC matrix yielded a more broadened peak, as depicted in Figure 2b. From the diffraction peak widening and peak intensity reduction, the dominance of amorphous region within a polymer system can be directly anticipated. It is well reported that the conductivity trend of the electrolytes can be verified through the analysis of XRD pattern [31]. However, theobtained results (Figure 2) show inadequate information regarding crystalline peaks. Therefore, the XRD data were deconvoluted and portrayed, as shown in Figure 3. Based on the results Figure 3a, CS:MC blend possesses crystalline peaks at2θ = 11.7°, 16.3°, 17.7°, 20.0° and 23.0°. As mentioned above, 2θ = 16.3° wasappointed to MC crystalline peak, while at 2θ = 11.7°, 17.7°, 20.0° and 23.0°wereassigned to CS. The appearances of more CS crystalline peaks weredue to the higher concentration of CS compared to MC. As observed in Figure 3b, the intensity of aforementioned five crystalline peaks weresuppressed with addition of 10 wt.% NH_4_SCN. These crystalline peaks wereobserved to become smaller when 40 wt.% NH_4_SCN was added. According to Hodge et al.’s criterion [32], a correlation between the intensity of the peak and the degree of crystallinity (χ_c_) has been established, in which the change in intensity and the broad nature of the XRD pattern are considered to be indicators for amorphous behavior of the sample. χ_c_ of the electrolyte can be calculated using the following equation,

(1) $$\chi_{C}=\frac{A_{c}}{A_{c}+A_{a}}$$

where *A_c_* and *A_a_* are the area of crystalline and amorphous peaks, respectively. The calculated values of χ_c_ are tabulated in Table 1. As shown in Table 1, χ_c_ reduced from 28.46 to 14.57 when 40 wt.% NH_4_SCN was included. This phenomenon shows that the addition of salt enhanced the amorphousness of the polymer blend. This result is harmonized with the reduction of crystalline peaks intensity in Figure 3. Here, upon the addition of the inorganic salt into the CS:MC system, the reduction of crystallinity was found to be related to the complex formation between polar groups of the polymer and cations of the salt due to electrostatic interactions, which retards the ordering of the crystalline regions within the polymer electrolyte by disrupting hydrogen bonding [24].

**Figure 4:**
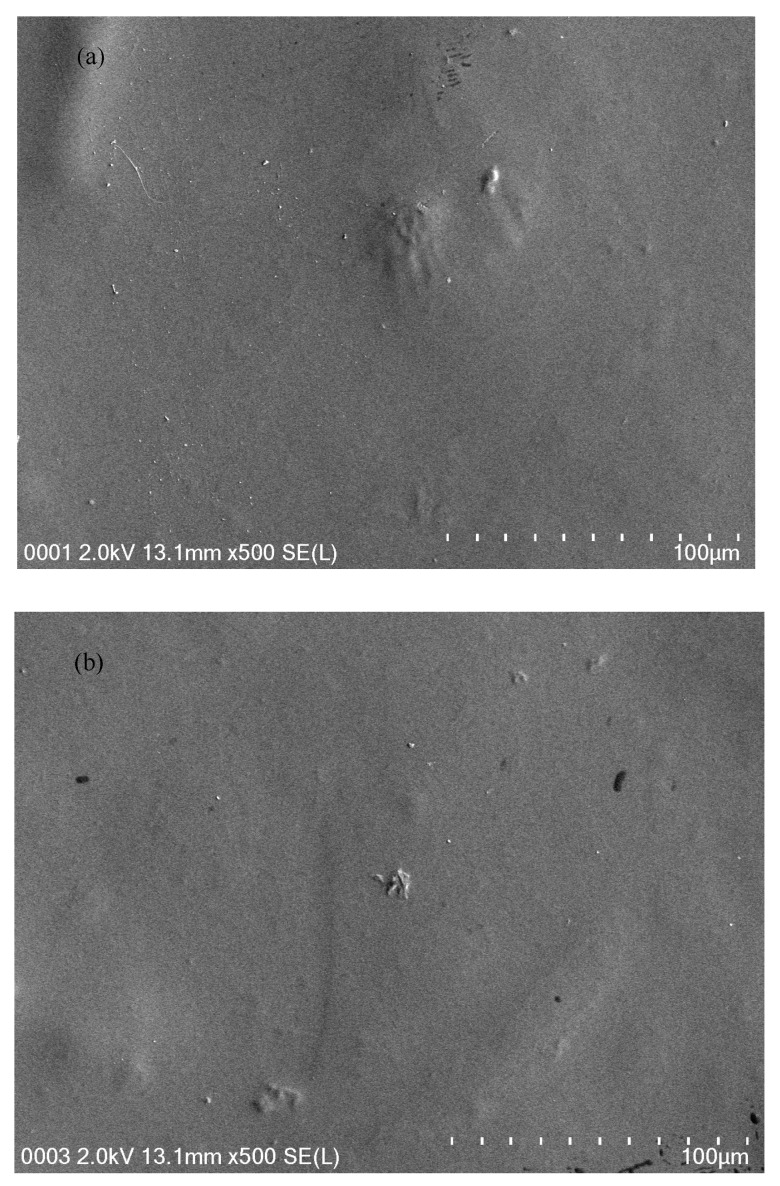

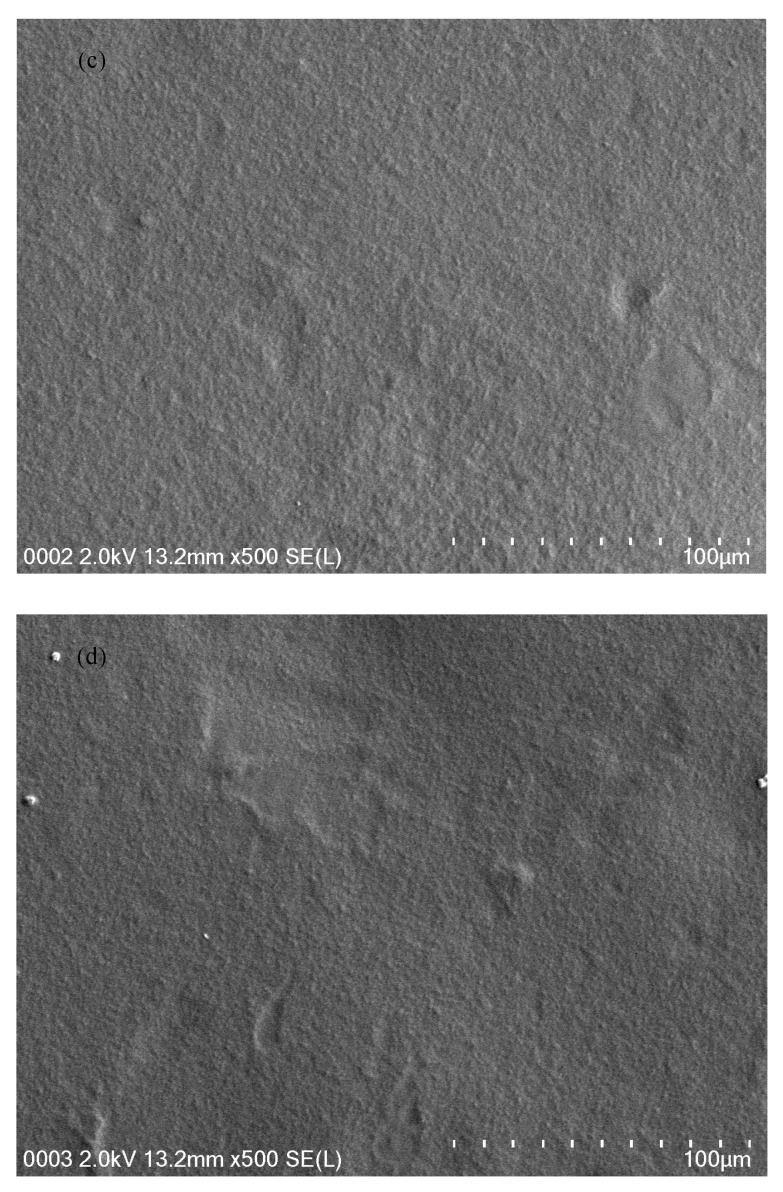
FESEM images for: (**a**) CSMCB1; (**b**) CSMCB2; (**c**) CSMCB3; and (**d**) CSMCB4.

To understand the compatibility between different components of the blend polymer electrolytes, detection of phase separations and interfaces using SEM has been commonly performed [30]. Figure 4a–d shows the SEM micrographs of the surfaces of CSMCB1, CSMCB2, CSMCB3 and CSMCB4 samples, respectively. As can be seen from the images, the surface of the samples was found to be smooth and homogenous with no evidence of any phase separation. It has been well reported that challenges still existed for obtaining well-defined morphologies and improving ion transport properties of polymer electrolytes.It is believed that high ion conductivity is associated with the smooth and homogenous surface appearances of the samples, meaning that it is related to the amorphous nature of the samples [33]. It has been demonstrated that the salt ions are capable of forming complexeswith polymer chains and reduce the hydrogen bonding interactions within the polymer matrix and thus increasing the amorphous content [34]. According to previous reports, joining of rough surfaces in polymer electrolytes has been assumed to act as the channels for ion conduction through the electrolyte [29,33,35]. In addition, particles appearance on the surface of samples was also referred to as ion-pairs formation, which does not participate in ionic conduction process [31,36,37]. The inter-ionic electrostatic forces and the incompatibility of the incorporated salt with the polymer can lead to salt aggregation at high salt concentration.As the salt content becomes larger than a critical value, the ion pairs are formed, leading to salt leakage to the surface [34]. This phenomenon results in an expected drop of conductivity, owing to the decreases in ion number. The surface morphology of the present samples in this work wasfound to be almost smooth and no protruding particles in high rate could be seen. Our findings indicate that our polymer blending is a new approach to fabricate polymer electrolytes with amorphous behavior even at high salt concentration. From the SEM micrograph images, it is obvious that little white specs appear on the surface of the samples. This phenomenon wasalso observed byBhad and Sangawar, who incorporatedPVA with NH_4_SCN salt [38]. They reported that the white particles observed on the electrolyte surface can act as paths or channels for proton conduction through the electrolyte.

### 3.2. Impedance Study

**Figure 5:**
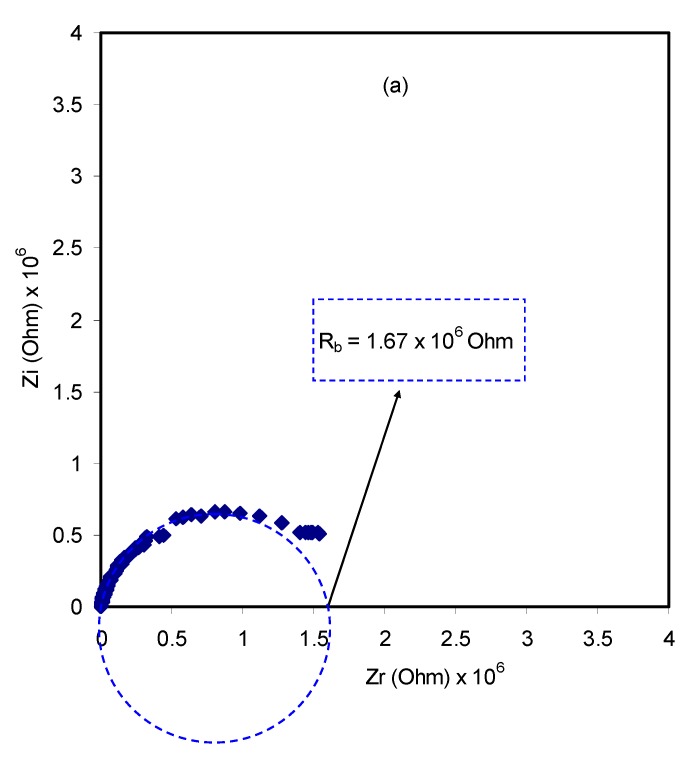

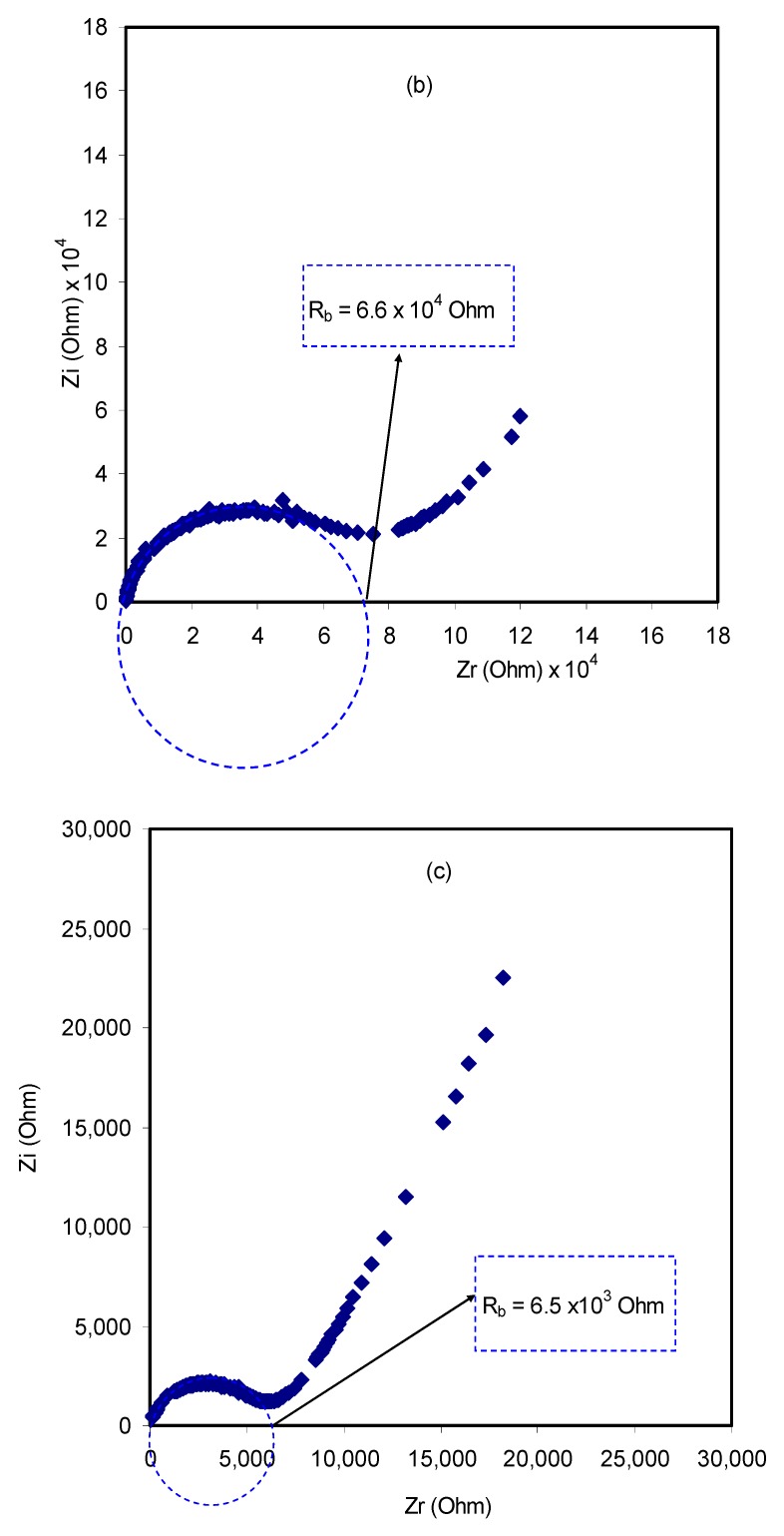

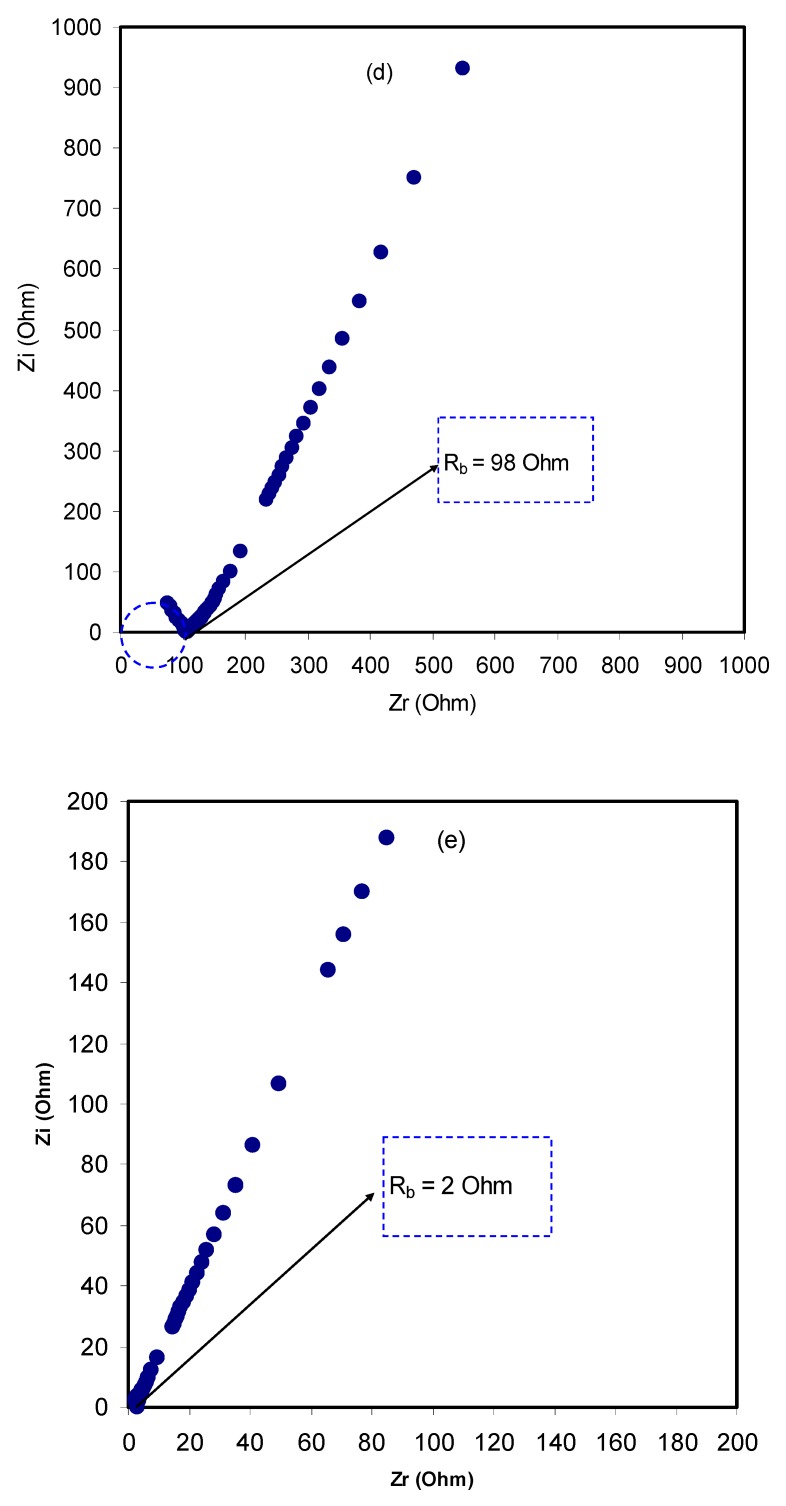
Impedance plots for: (**a**) CSMCB0; (**b**) CSMCB1; (**c**) CSMCB2; (**d**) CSMCB3; and (**e**) CSMCB4 blend electrolyte samples.

**Figure 6:**
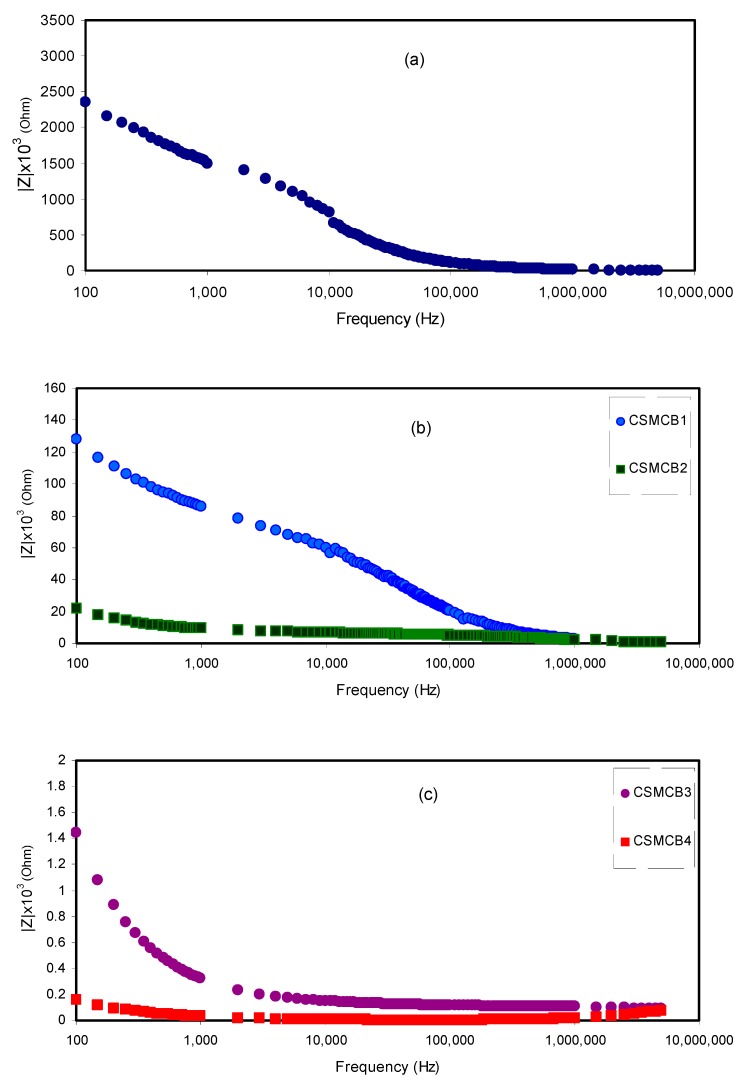
Bode plots for: (**a**) pure CS:MC blend film; (**b**) CSMCB1 and CSMCB2; and (**c**) CSMCB3 and CSMCB4 blend electrolyte samples.

It is well known that ionic conductivity of the polymer based on ion conductors mainly depends on the concentration of ion carriers and their mobility. For this purpose, impedance spectroscopy has been found to be a simple and powerful technique to study the electrical properties of polymer-based electrolytes [39]. Figure 5 shows the impedance plots of CSMCB0, CSMCB1, CSMCB2, CSMCB3 and CSMCB4 samples. It is clear that the pure CS:MC (CSMCB0) sample exhibits almost a perfect semicircle shape. The distinctive plots of blend electrolyte samples that contain ammonium salt from 10 to 30 wt.% of NH_4_SCN consist of a high frequency semicircle followed by a low frequency straight line. The high frequency semicircle presents the bulk conductance, which is equivalent to the parallel combining of bulk resistance (*R_b_*) and bulk capacitance (constant phase element, CPE) of the polymer electrolytes [31]. It can be noted that, at low frequency, there are traces related to electrode polarization [40,41,42], which is a characteristic of a diffusion process [43]. From the intercept of the straight line on the *Z_r_* axis, one can obtain the bulk resistance of the electrolyte (*R_b_*). The ionic conductivities were calculated through the relation of *σ* = *l/R_b_A*, where *l* is the thickness, *R_b_* is bulk resistance and *A* is the known area of the electrolyte film. The DC conductivity values are presented in Table 2. It can be seen in Table 2 that the sample incorporated with 40 wt.% of NH_4_SCN exhibits the highest DC conductivity. Moreover, the exceeding interpretation and anticipating semicircles for Nyquist plots are further supported by examining the Bode plots. From the perspective of electrochemical, Bode plots are particularly useful in understanding the charge transfer process in electrolyte materials [44]. Figure 6 shows the Bode plots for the blend electrolyte films at room temperature. Previous studies have shown that three distinguished regions, namely capacitive, diffusion and charge transfer regions should be exhibited from the Bode plots [44,45,46]. The capacitive region (plateau region) can typically be observed at a very low frequency, from 10^−2^ to 100 Hz. However, in our study, this region could not be examined due to the frequency limitations of our measuring equipment. As discussed for the impedance plots in Figure 5, the semicircle has been related to the ion transfer in amorphous phase of electrolytes and the tails refer to the contribution of Warburg or diffusion of ions and thus their accumulation at the electrode/electrolyte interface [39,41,44]. The accumulation of ions at both sides of the electrolyte will produce electrical double layer capacitances. The results clearly indicate that, with increasing salt concentration from 10 to 40 wt.%, the Warburg contribution (tail regions) increased and hence the resistance decreased, owing to the large amount of carrier density. It is clear in Figure 6a that pure CS:MC films exhibit high charge transfer resistance. Obviously, with increasing salt concentrations, as shown in Figure 6b,c, the charge transfer resistance decreased. The low frequency dispersion region in Bode plots ascribed to ion diffusion phenomena and the high frequency region is attributed to charge transfer resistance. In Figure 5 and Figure 6, it is clear that the samples incorporated with 40 wt.% of NH_4_SCN exhibit the lowest charge transfer resistance and thus a high DC conductivity resulted. Thus, the Bode plots also support the results estimated from impedance plots. From the physics point of view, it is essential to prepare polymer electrolytes with high DC conductivity and, from the chemistry viewpoint, it is important for the samples to have the low charge transfer resistance.

**Figure 7:**
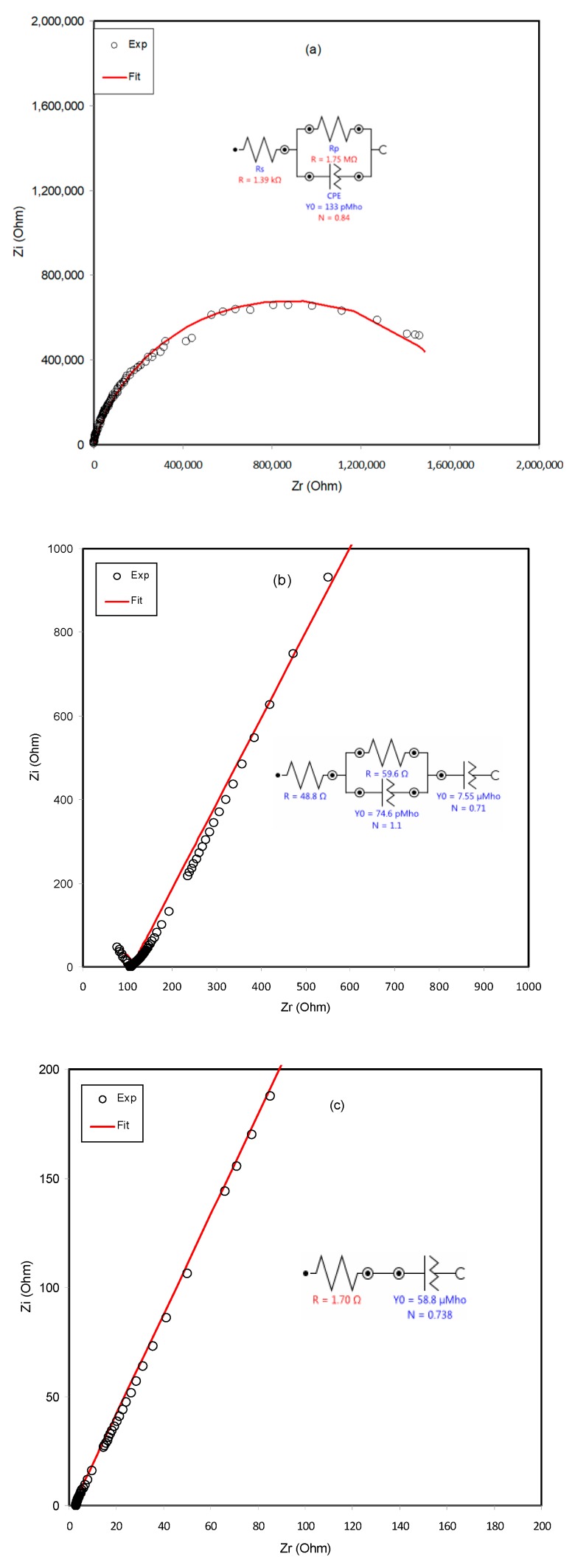
Experimental Impedance and EEC fitting plots for: (**a**) CSMCB0; (**b**) CSMCB3; and (**c**) CSMCB4 blend electrolyte samples.

The electrical equivalent circuit (EEC) model was conducted for selected samples. Typically, EEC is used for the analysis of impedance spectroscopy given the fact that the model is simple, quick and provides a comprehensive picture of the system [47]. From the EEC modeling, the resistance and circuit elements associated with the electrical properties of the samples can be obtained. Figure 7a–c shows the fitting of impedance plots with EEC model for selected samples. It can be seen that the impedance of pure CS:MC can only be represented by a parallel combination of resistance and CPE (see the inset), and thus it can be considered as nearly insulator with high resistivity. The samples incorporated with 30 wt.% of NH_4_SCN salt show incomplete semicircle at high frequency region and exhibit a spike at low frequency data points. The high frequency region demonstrates the association of R_b_ and CPE, whereas the low frequency region shows CPE, i.e., the developed double-layer capacitance between the electrodes and the blend electrolyte. Generally, the term CPE is usually used in equivalent circuits rather than ideal capacitors in real system because the behavior of real SPE is different from that of an ideal capacitor engaged in a pure semicircular pattern [48]. The depressed semicircle can be explained through the CPE rather than a capacitor [49,50,51], and the low frequency tail is introduced as another CPE. It is interesting that, at 40 wt.% of NH_4_SCN salt, only a spike can be observed, which is a characteristic for diffusion process. In the present case, the EEC can be shown by a combination of R_b_ and CPE in series [50,51], as shown by the inset of Figure 7c.

The impedance of Z_CPE_, which appears as parallel combination in EEC model for pure CS:MC, can be written as [49,50,51]:

(2) $$Z_{CPE}=\frac{\cos(\pi n/2)}{Y_{m}\omega^{n}}-j\frac{\sin(\pi n/2)}{Y_{m}\omega^{n}}$$

where Y_m_ indicates the CPE capacitance, ω is the angular frequency and *n* is related to the deviation of the vertical axis of the plot within the complex impedance plots. Finally, the real (Zr) and imaginary (Zi) values of complex impedance (Z*) related with the equivalent circuit (insets of Figure 7a) can be expressed as [51]:

(3) $$Z_{r}=R_{s}+\frac{R_{1}+R_{1}^{2}Y_{1}\omega^{n1}\cos(\pi n_{1}/2)}{1+2R_{1}Y_{1}\omega^{n_{1}}\cos(\pi n_{1}/2)+R_{1}^{2}Y_{1}^{2}\omega^{2n_{1}}}$$

(4) $$Z_{i}=\frac{R_{1}^{2}Y_{1}\omega^{n_{1}}\sin(\pi n_{1}/2)}{1+2R_{1}Y_{1}\omega^{n_{1}}\cos(\pi n_{1}/2)+R_{1}^{2}Y_{1}^{2}\omega^{2n_{1}}}$$

In a Cole–Cole plot, at a certain high salt concentration of the salt (see Figure 7b), the incomplete semicircle with a spike can be separated. That is, two constant phase elements are required to fit the experimental data points, one is in parallel and the other, due to tail, is in series. The real (*Z_r_*) and imaginary (*Z_i_*) values of complex impedance (Z*) related with the equivalent circuit (insets of Figure 7b) can be expressed as [49,50]:

(5) $$Z_{r}=R_{s}+\frac{R_{1}+R_{1}^{2}Y_{1}\omega^{n1}\cos(\pi n_{1}/2)}{1+2R_{1}Y_{1}\omega^{n_{1}}\cos(\pi n_{1}/2)+R_{1}^{2}Y_{1}^{2}\omega^{2n_{1}}}+\frac{\cos(\pi n_{2}/2)}{Y_{2}\omega^{n_{2}}}$$

(6) $$Z_{i}=\frac{R_{1}^{2}Y_{1}\omega^{n_{1}}\sin(\pi n_{1}/2)}{1+2R_{1}Y_{1}\omega^{n_{1}}\cos(\pi n_{1}/2)+R_{1}^{2}Y_{1}^{2}\omega^{2n_{1}}}+\frac{\sin(\pi n_{2}/2)}{Y_{2}\omega^{n_{2}}}$$

In a Cole–Cole plot at 40 wt.% of NH_4_SCN salt, as shown in Figure 7c, the semicircle disappears, indicating that only the resistive component of the polymer is predominated [52]. In this case, the values of *Zr* and *Zi* associated to the EEC can be expressed as:

(7) $$Z_{r}=R+\frac{\cos(\pi n/2)}{Y_{m}\omega^{n}}$$

(8) $$Z_{i}=\frac{\sin(\pi n/2)}{Y_{m}\omega^{n}}$$

### 3.3. EDLC Study

#### 3.3.1. TNM and LSV Study

**Figure 8:**
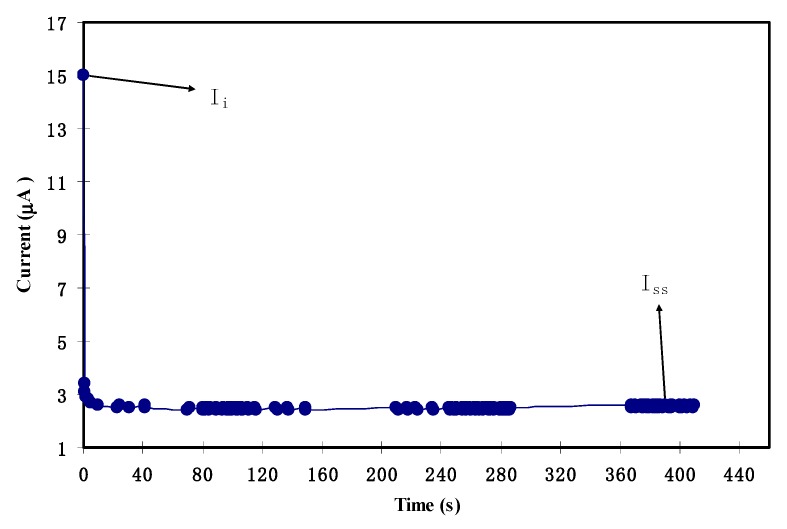
Current with respect to time for the cell assembled with stainless steel/[CS:MC]:NH_4_SCN/stainless steel at an applied potential (∆V) of 0.20 V at ambient temperature.

To use the polymer electrolyte for application, it is essential to study the TNM and LSV. In polymer electrolyte, it is crucial to determine the dominant charge carrier species which can be done using transference number analysis (TNM). The ion (*t_ion_*) and electron (*t_el_*) transference number can be determined via the equation:

(9) $$t_{ion}=\frac{I_{i}-I_{ss}}{I_{i}}$$

(10) $$t_{ion}=1-t_{el}$$

where *I_ss_* is the steady-state current and *I_i_* is the initial current. Figure 8 depicts the polarization of the electrolyte at 0.20 V. *I_i_* was observed at 15 μA. The large value of initial current is due to contribution of both ion and electron at the initial stage. As the electrodes used werestainless steel, which is known to block ions, a drastic drop of current wasnoticed before constant at 2.4 μA [52]. This phenomenon portrays the behavior of an ionic conductor [53]. Ions werefound to be the dominant charge carriers in the polymer electrolyte as *t_ion_* was 0.84 while *t_el_* was 0.16. 

**Figure 9:**
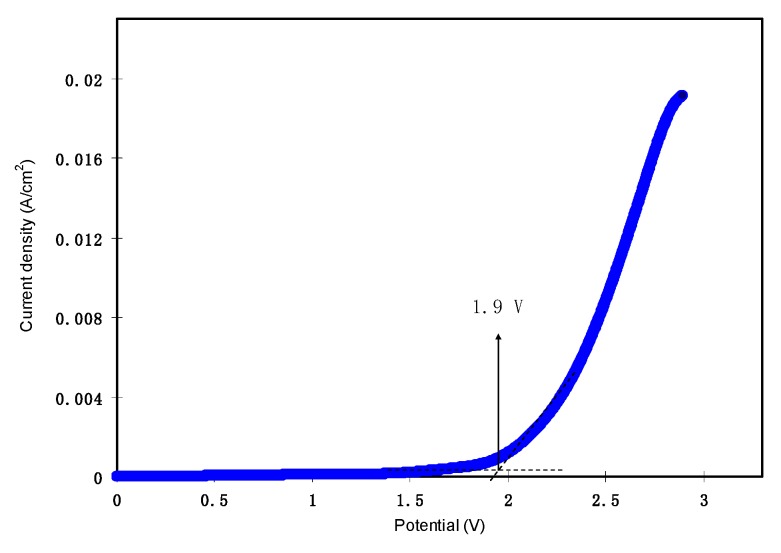
LSV curve for the cell with stainless steel/[CS:MC]:NH_4_SCN/stainless steel at scan rateof 50 mV/s at ambient temperature.

Working potential range and electrochemical stability of the polymer electrolyte is an essential criterion to be determined for its application in electrochemical devices. LSV was conducted to test the electrochemical stability of the highest conducting chitosan-methycellulose-NH_4_SCN system. Figure 9 illustrates the LSV plot of the highest conducting chitosan-methycellulose-NH_4_SCN at room temperature. No obvious current was recorded in the range 0–1.8 V. As the potential exceeded 1.9 V, the current was observed to gradually increase. The increment in current corresponded to the decomposition of the polymer electrolyte [54]. This indicates that chitosan-methycellulose-NH_4_SCN system is electrochemically stable up to 1.9 V. According to Noor and Isa [55], the electrochemical stability for carboxymethylcellulose-NH_4_SCN system is 1.6 V. It is well known that the standard electrochemical window is approximately 1 V for protonic devices [56]. Hence, the highest conducting electrolyte for chitosan-methycellulose-NH_4_SCN system can be employed as electrodes separator in proton-based energy devices.

#### 3.3.2. CV and EDLC Characteristics

**Figure 10:**
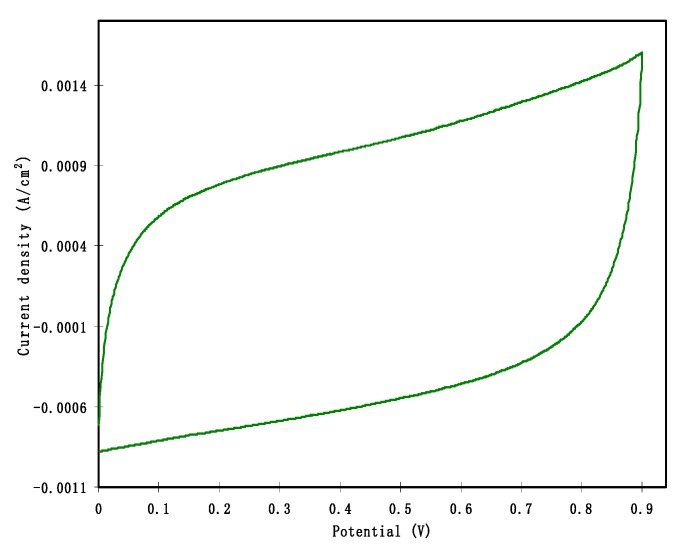
Plot of the assembled EDLC at 10 mV/s from 0 to 1 V.

The cyclic voltammetry (CV) for the fabricated EDLC at 10 mV s^−1^ is shown in Figure 10. CV measurement is based on potentiodynamic electrochemical measurement. This analytic technique involves the electrode potential determination corresponding linearly to the time. In other words, the electrode potential is plotted linearly versus time. In addition, the current versus applied voltage related to the working electrode is plotted, which is called the cyclic voltammogram trace [57]. The CV plot was observed as a leaf shape, which is close enough to a perfect rectangular. Such characteristic reveals that the fabricated supercapacitor cell illustrates EDLCs behavior and is suitable for EDLC application. An insignificant role from electrons was confirmed by the nonexistence of redox peak, which is indicative of non-Faradic process involved in fabricated supercapacitor, hence confirming its EDLCs behavior [58]. This is due to the porous structure of activated carbon as well as internal resistance [59]. In addition, the CV plot possesses no redox peak, indicating the occurrence of charge double-layer at the surface of activated carbon electrodes [60]. The specific capacitance (*C_spe_*) of the EDLC can be determined from the CV plot using the following equation:

(11) $$C_{spe}=\frac{\int_{V_{1}}^{V_{2}}I\left(V\right)dV}{2m\left(V_{2}-V_{1}\right)\left(\frac{dV}{dt}\right)}$$

where $\int_{V_{1}}^{V_{2}}I\left(V\right)dV$ is the area of the CV plot which is obtained from OriginPro 8.5 software, *m* is the activate material mass (activated carbon), (*V*_2_ − *V*_1_) is the potential range and $\frac{dV}{dt}$ is the scan rate [61]. The *C_spe_* for the fabricated EDLC was found to be 66.3 F g^−1^. This value was compared to the *C_spe_* from charge–discharge analysis.

**Figure 11:**
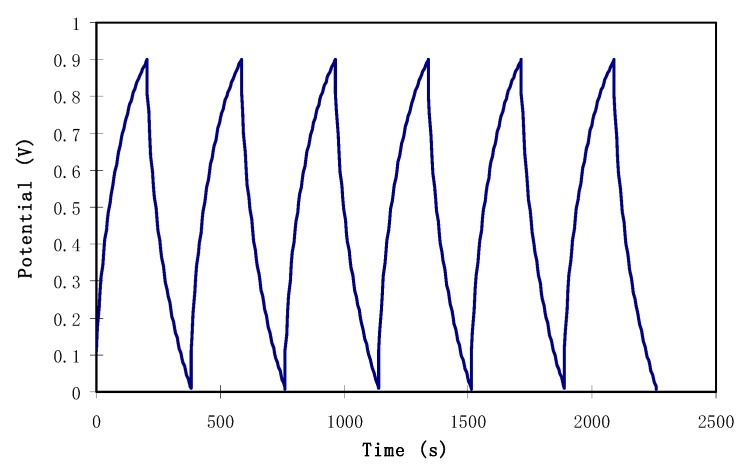
Galvanostatic charge–discharge performances of the EDLC cell over the first sixcycles.

**Figure 12:**
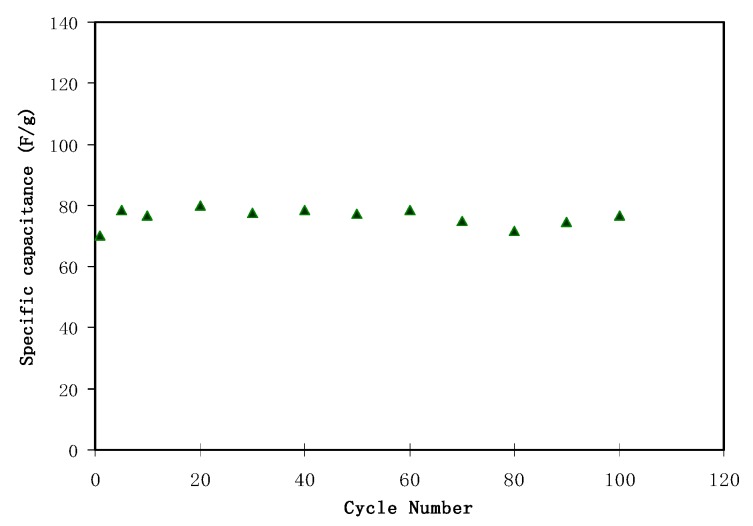
Specific capacitance (Cspe) of the assembled EDLC up to the 100th cycle.

The rechargeability of the fabricated EDLC at 0.4 mA cm^−2^ for the selected cycle is shown in Figure 11. It was observed that the discharge slope is almost linear, signifying the existence of capacitive behavior at the surface of the electrodes [62]. As the slope of discharge curve is given as (*x*), the *C_spe_* of EDLC can be obtained from the following equation:

(12) $$C_{spe}=\frac{i}{xm}$$

where *i* is the applied current. Figure 12 shows the *C_spe_* versus cycle number. The value of *C_sp_* at the first cycle was found to be 69.9 F g^−1^. The *C_spe_* value obtained from CV analysis was 66.3 F g^−1^, which is 5.1% lower than the one obtained from charge–discharge analysis. Therefore, the *C_spe_* value of the EDLC in this work is considered to be reliable. At the fifth cycle, the *C_spe_* increased to 78.4 F g^−1^ and continued to be constant at an average value of 76.7 F g^−1^ up to the 100th cycle. The specific capacitance achieved in the present work is of great interest compared to the low specific capacitance values of 2.6–3.0 and 1.7–2.1 F g^−1^ reported for the EDLC cells with the Mg- and Li-based PEO polymer electrolytes incorporated with ionic liquids [63]. Moreover, the maximum *C_spe_* obtained in the current work is found to be even greater than that obtained (61.7 F g^−1^) for ionic liquid-based gel polymer electrolyte reported by Mukta Tripathi and S.K. Tripathi [64]. The specific capacitance of the current work is also comparable to those reported for gel based polymer electrolytes by Boonen et al. [65] and Łatoszyńska et al. [66], which were about 87.3 F/g and 90 F/g, respectively. Consequently, polymer blending can be regarded as a novel approach to fabricate EDLC with high specific capacitance at ambient temperature. The results of the present work may present new knowledge for EDLC fabrication-based natural biopolymers incorporated with proton ion conductors. 

The equivalent series resistance (*R_esr_*) is another crucial parameter that needs to be identified to study the internal resistance of the EDLC. *R_esr_* of the EDLC can be expressed as:

(13) $$R_{esr}=\frac{V_{d}}{i}$$

**Figure 13:**
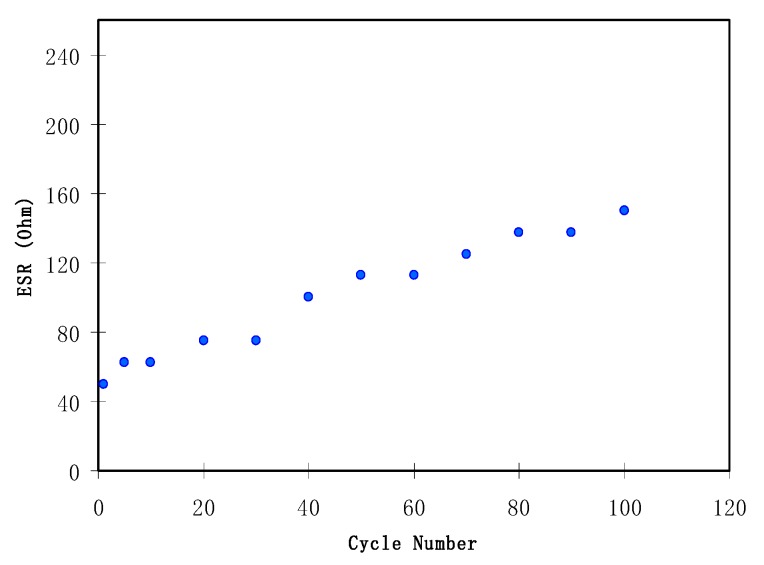
Series resistance (*R_esr_*) of the assembled EDLC up to the 100th cycle.

As observed in Figure 11, there wasa small voltage drop (*V_d_*) before each discharging process. The voltage drop was found to be in the range 0.04–0.12 V, which could be due to the internal resistance within EDLC. Figure 13 shows *R_esr_* of EDLC for 100 cycles. The value of *R_esr_* of EDLC ranges from 50.0 to 150.0 Ω. Resistance of electrolyte, current collectors and current collector–electrolyte gap are the reasons for internal resistance existence [67]. Asmara et al. [68] stated that small value of *R_esr_* portrays good electrode–electrolyte contact, which indicates that it is easy for ions to migrate toward the surface of the electrolyte to form electrical double-layer. It is noticeable that the *R_esr_* of the EDLC increased with positive slope throughout the 100 cycles. At the first cycle, *R_esr_* of the EDLC was 50.0 Ω and increased to 100.0 Ω at the 40th cycle. As the cycle number increased to 100, *R_esr_* also elevated to 150.0 Ω. Wang et al. [69] reported the same pattern where *R_esr_* increased with positive slope while the *C_spe_* was constant up to 1000 cycle. According to the report by Shukur et al. [70], *R_esr_* of EDLC continues to increase with constant value of *C_spe_* for starch-lithium acetate electrolyte system. The authors also stated that *R_esr_* is related to the power density of EDLC. The increment in *R_esr_* value is due to electrolyte depletion. Fast charging and discharging processes cause free ions to recombine and develop ion pairs/aggregates, leading to ionic conductivity reduction. Kang et al. [71] also stated that *R_esr_* is strongly related to the conductance of the electrolyte.

**Figure 14:**
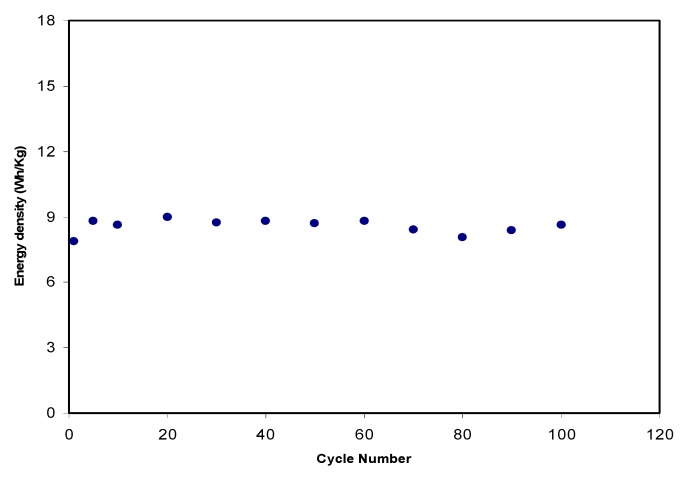
Density (*E_d_*) of the assembled EDLC up to the 100th cycle.

The energy density (*E_d_*) of the fabricated EDLC can be calculated via the equation:

(14) $$E_{d}=\frac{C_{s}V}{2}$$

where *V* = 1 V. Figure 14 shows *E_d_* of the fabricated EDLC as a function of the number of cycles. As shown in the figure, *E_d_* at the first cycle was7.88 Wh kg^−1^ and then increased to 8.81 Wh kg^−1^. Then, in the following steps, from the 10th to 100th cycles, *E_d_* stabilized at an average value of 8.63 Wh kg^−1^. Hence, it can be assumed that, from the 10th to 100th cycle, the transportation of ion possesses almost the same energy barrier [12]. The energy density (8.63 Wh/Kg) achieved for the EDLC cell in the present work is of great interest compared to that reported (5.5 Wh/Kg) by Mukta Tripathi and S.K. Tripathi [64] for ionic liquid-based gel electrolytes.

The power density (*P_d_*) of the EDLC is dependent on the square of the maximum voltage denoted by *V* and equivalent series resistance denoted by *R_esr_* as shown in the following equation:

(15) $$P_{d}=\frac{V^{2}}{4mR_{esr}}$$

**Figure 15:**
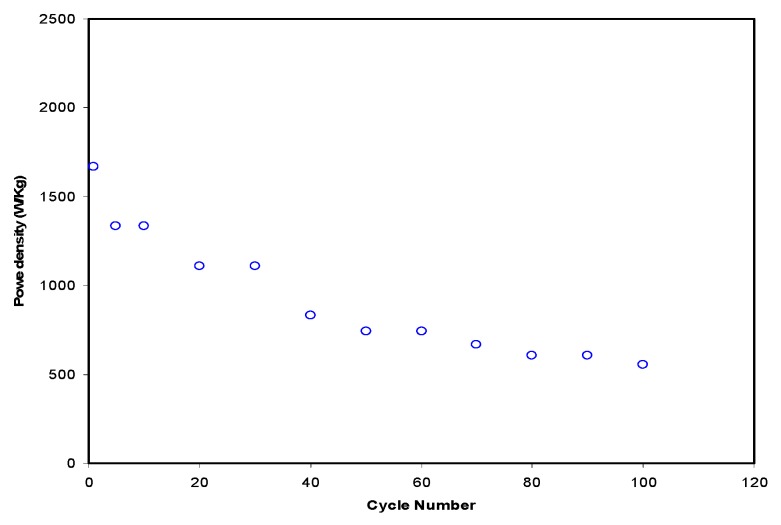
Power density (Pd) of the assembled EDLC up to the 100th cycle.

The ESR is the overall sum of resistances corresponding to electrode, electrolyte and the diffusion resistances of ions in electrode pores [57]. The calculated values of *P_d_* are shown in Figure 15. The first cycle had *P_d_* of 1666.6 W kg^−1^. The *P_d_* dropped to 747.0 W kg^−1^ at the 50th cycle and continued to drop to 555.5 W kg^−1^ as the EDLC completed 100 cycles. The same power density reduction pattern has been reported in other studies [61,70]. The pattern of power density is in accordance with the trend of *R_esr_*. The drops in *P_d_* value were due to the re-association of cation and anion to form ion aggregation during rapid charge–discharge process, which obstructs the ionic transport [72]. According to recent study established by Coromina et al., [73], the energy stored in EDLC containing aqueous H_2_SO_4_ or ionic liquid electrolytes can be delivered at power densities >1 kW kg^−1^. Thus, the performance of these devices bridges the performance gap between those of EDLCs and batteries. Thus, the results of the current work are crucial to fabricate promising EDLC cells with high power density using biopolymer-based blend electrolytes. Apart from the numerical parameters, both energy and power densities are explicitly reliant on mass loading of active material. It is established that there is an inverse reliance of energy and power density on mass loading. Additionally, the low mass loading and relatively low current are reported to be responsible for providing enhanced electrochemical performance [57].

## 4. Conclusions

The present work presents a promising EDLC based on CS:MC polymer blends incorporated with proton conducting inorganic salt with large specific capacitance (76.7 F g^−1^) and energy density (8.63 Wh kg^−1^). The change in intensity and the broad nature of the XRD pattern of the doped samples compared to the pure CS:MC system were ascribed to disruption of hydrogen bonding in polymer blend electrolyte samples. The morphology of the samples appearing in FESEM images is almost dark and smooth, supporting the amorphous behavior of the solid electrolyte films. The impedance and Bode plot results indicate that the charge transfer resistance decreased with increasing salt concentration due to the increase of charge carrier concentration. EEC model was conducted for selected samples to explain the complete picture of the electrical properties. The performance of EDLC cells was examined by impedance spectroscopy, cyclic voltammetry and constant current charge–discharge techniques at room temperature. It was observed that the proton-based polymer electrolyte exhibits good performance as electrolyte for EDLCs application. Ions were found to be the dominant charge carriers in the polymer electrolyte as the *t_ion_* obtained was0.84 while the *t_el_* was 0.16. The LSV investigation indicated that chitosan-methycellulose-NH_4_SCN system is electrochemically stable up to 1.8 V. The CV plot exhibits no redox peak, indicating the occurrence of charge double-layer at the surface of activated carbon electrodes. The *C_spe_* for the fabricated EDLC was found to be 66.3 F g^−1^. The value of *C_sp_* at the first cycle was 69.9 F g^−1^. The value of *C_spe_* obtained from CV analysis was 5.1% lower than the one from charge–discharge analysis. Thus, the value of *C_spe_* of the EDLC in this work was considered to be reliable. The *R_esr_* of the EDLC ranged from 50.0 to 150.0 Ω. *P_d_* was obtained to be 1666.6 W kg^−1^ at the first cycle and then dropped to 747.0 W kg^−1^ at the 50th cycle. 

## Figures and Tables

**Table 1:** Degree of crystallinity of each polymer blends.

Polymer Electrolyte Blend	Degree of Crystallinity (χ_c_)
CS:MCB0	28.46
CSMCB1	22.14
CSMCB2	20.38
CSMCB3	16.03
CSMCB4	14.57

**Table 2:** Conductivity for pure CS:MC and blend electrolyte films at room temperature.

Sample Designation	DC conductivity (S/cm)
CSMCB0	3.35 × 10^−9^
CSMCB1	8.49 × 10^−8^
CSMCB2	8.62 × 10^−7^
CSMCB3	5.71 × 10^−5^
CSMCB4	2.80 × 10^−3^
